# Association Analysis of *BRCA1*, *PPAP2B*, and *KCNN3* Gene Polymorphisms With Litter Size in Sheep

**DOI:** 10.1155/vmi/2771616

**Published:** 2025-12-28

**Authors:** Yu He, Xiangyu Wang, Chen Liang, Mingxing Chu

**Affiliations:** ^1^ State Key Laboratory of Animal Biotech Breeding, Institute of Animal Science, Chinese Academy of Agricultural Sciences, Beijing 100193, China, caas.cn; ^2^ College of Animal Science, Shanxi Agricultural University, Taigu, Shanxi, 030801, China, sxau.edu.cn

**Keywords:** *BRCA1*, *KCNN3*, litter size, *PPAP2B*, sheep, SNPs

## Abstract

**Background:**

Reproductive efficiency is a key economic trait in sheep production, with litter size being a major determinant of productivity. Genetic polymorphisms in candidate genes such as *BRCA1*, *PPAP2B*, and *KCNN3* may influence fecundity, but their association with sheep prolificacy remains to be fully elucidated. Identifying genetic markers linked to high litter size could enhance breeding strategies for improved reproductive performance.

**Objective:**

This study aimed to (1) investigate the relationship between *BRCA1*, *PPAP2B*, and *KCNN3* gene polymorphisms and sheep litter size and (2) identify potential genetic markers for high fecundity in sheep.

**Methods:**

Whole‐genome resequencing combined with Sequenom MassARRAY SNP genotyping was used to analyze five polymorphic loci in *BRCA1*, *PPAP2B*, and *KCNN3* across five sheep breeds, including high‐prolificacy breeds (Small‐Tailed Han sheep, Hu Sheep, and Cele Black Sheep) and low‐prolificacy breeds (Sunite sheep and Bamei mutton sheep). Small‐Tailed Han sheep were selected for association analysis with litter size. Chi‐square tests assessed Hardy–Weinberg equilibrium (HWE), and association studies evaluated genotype effects on reproductive traits.

**Results:**

BRCA1 gene polymorphisms (g.42581575 T > C, g.42560830 A > T, g.42548851 C > T) exhibited significant differences (*p* < 0.01) in genotype and allele frequencies between high‐ and low‐prolificacy breeds. All three loci were in HWE (*p* > 0.05) except in Hu sheep. The *KCNN3* g.103522805 C > A locus also showed significant frequency differences (*p* < 0.01) between breeds but deviated from HWE in Bamei mutton sheep (*p* < 0.05).Only the *PPAP2B* g.77092130 G> A locus demonstrated a significant association (*p* > 0.05) with litter size in Small‐Tailed Han sheep, where the GG genotype was more frequent than the AA genotype in the second parity.

**Conclusion:**

This study identified *BRCA1*, *PPAP2B*, and *KCNN3* as potential genetic markers influencing sheep litter size. The *BRCA1* and *KCNN3* polymorphisms showed strong breed‐specific associations, while the PPAP2B GG genotype may favor higher fecundity in Small‐Tailed Han sheep. These findings provide valuable insights for marker‐assisted selection in sheep breeding programs aimed at improving reproductive efficiency. Further functional studies are needed to validate the biological mechanisms underlying these genetic associations.

## 1. Introduction

Reproductive performance stands as one of the most economically significant traits in modern sheep production systems [1], with fecundity parameters including estrous cyclicity, breeding seasonality, and ovulation rate serving as critical determinants of flock profitability [2]. The inherent variability in these physiological traits, particularly in ovulation rate, creates substantial differences in production efficiency across breeds and individuals [3]. Among reproductive metrics, litter size emerges as the most direct indicator of reproductive success, representing a complex quantitative trait influenced by an intricate interplay of genetic predisposition, nutritional status, and management practices [4]. Within this multifactorial framework, ovulation rate has been identified as the primary biological determinant influencing lamb output potential [5], making its genetic regulation a focal point for reproductive enhancement strategies.

At the molecular level, the bone morphogenetic protein receptor 1B (BMPR1B) gene has been established as a major genetic regulator of ovine reproductive performance [6]. Located on chromosome 6, this gene exerts pleiotropic effects on multiple aspects of female reproduction, including follicular development, ovulation rate modulation, and consequent litter size determination [7]. The gene’s activity in ovarian tissue is significantly influenced by *BRCA1* [8], a transcription factor that binds to specific regulatory elements within the BMPR1B promoter region [9]. This molecular interaction enhances BMPR1B transcriptional activity, promotes granulosa cell survival, and ultimately increases the ovulation quota, highlighting the functional importance of this regulatory axis in determining ovine reproductive potential [9].

A persistent challenge in global sheep production systems stems from the pronounced seasonality [10] of reproductive activity observed in most breeds. This biological constraint results in uneven lamb availability throughout the year, creating significant limitations for consistent meat production and market supply. While the majority of Chinese indigenous sheep breeds exhibit strict seasonal breeding patterns conforming to photoperiodic regulation, Hu sheep represent a notable exception with their demonstrated capacity for continuous, nonseasonal reproductive activity [11, 12]. This unique biological characteristic suggests the presence of distinct genetic mechanisms governing reproductive physiology in this breed, with *PPAP2B* emerging as a particularly promising candidate gene. As a member of the evolutionarily conserved lipid phosphate phosphatase family, *PPAP2B* encodes the Lpp3/Pap2b enzyme that plays fundamental roles in cellular growth regulation and developmental processes [13, 14]. The gene’s dynamic expression pattern during reproductive cycles and its elevated expression specifically in Hu sheep ovarian tissue during estrus strongly implicate its involvement in mediating nonseasonal reproductive capability.

The genetic regulation of reproductive success extends beyond conception and ovulation to include critical parturition processes. In this context, potassium channel genes, particularly *KCNN3* encoding calcium‐activated potassium channels, have attracted research attention for their potential role in regulating uterine function [15]. While these channels have been well characterized in other mammalian species for their crucial involvement in modulating myometrial contractility during pregnancy and labor, their specific functions in ovine reproduction remain largely unexplored. Emerging evidence suggests these ion channels may influence reproductive success through multiple mechanisms [16], including regulation of myometrial excitability and modulation of cervical ripening processes [17]. The potential involvement of *KCNN3* in ovine parturition physiology presents an important avenue for investigation, as understanding its regulatory mechanisms could yield novel insights for improving reproductive management and lamb survival rates [18].

This comprehensive investigation focuses on elucidating the genetic architecture underlying the exceptional reproductive performance of sheep, with particular emphasis on characterizing the functional roles and interactions of three key candidate genes: BMPR1B, *PPAP2B*, and *KCNN3* [1]. Through systematic examination of these genetic factors and their associated molecular pathways, this research aims to advance our understanding of the biological mechanisms governing prolificacy in sheep [19]. The findings are expected to contribute significantly to the development of targeted genetic improvement strategies, ultimately enhancing the efficiency and sustainability of sheep production systems to meet growing global demands for sheep products. By integrating traditional breeding approaches with modern molecular genetic insights, this study seeks to bridge the gap between fundamental reproductive biology and practical applications in sheep husbandry.

## 2. Materials and Methods

### 2.1. Experimental Samples

Among the multilamb sheep, 384 Small‐Tailed Han sheep were from Shandong, 96 Hu sheep were from Xuzhou, Jiangsu, and 96 Cele black sheep were from Cele, Xinjiang; among the single‐lamb sheep, 96 Sunite sheep were from Bayannaoer Ulat Qianqi and 96 Bamei mutton sheep were from Bayannaoer Linhe District.

### 2.2. Blood DNA Extraction

Blood was collected from the jugular vein of all sheep and anticoagulated with glucose citrate treatment and stored at −20°C. The respective lambing litters and lambing numbers of the Small‐Tailed Han sheep were also collected.

### 2.3. Primer Design and Genotyping

The *PPAP2B* gene g.77092130 G > A locus and the *KCNN3* gene g.103522805 C > A locus and the *BRCA1* gene g.42548851 C > T, g.42581575 T > C and g.42560830 A > T loci were genotyped using the Sequenom MassARRAY SNP technology in single‐ and multilamb genotyping in single and multiple lamb breeds of sheep. The single‐base extended primers for three loci were designed via MassARRAY Assay Design v. 3.1 based on the sheep sequences of *PPAP2B*, *BRCA1* and *KCNN3* available in GenBank ARS‐UI_Ramb_v3.0 (accession no.: XM_012154685.5, XM_060395679.1, and XM_027975753.3). The amount of DNA required for a single typed sample was 20 μL, with DNA concentrations ranging from 40 to 80 ng/μL. The genomic DNA of the samples was amplified using the amplification primers shown in Table 1.

**Table 1:** Primer information in sheep *PPAP2B*, *BRCA1*, and *KCNN3* genes.

Gene name	Primer sequence	Tm (°C)
PPAP2B	F: ACGTTGGATGTTTACCACGGAGGAAGTCTG R: ACGTTGGATGCCGCTGTGAAGAAATCTAGG	49.8
BRCA1‐1	F: ACGTTGGATGAACCATCAAGGCCCAAAACG R: ACGTTGGATGGGGCTTTTTGTCAACACTGG	50.7
BRCA1‐2	F: ACGTTGGATGCTAGGTTCTCCTCTGTTTCC R: ACGTTGGATGCTAGGTTCTCCTCTGTTTCC	50.5
BRCA1‐3	F: ACGTTGGATGTGAGGGAAGAAAGTGTGAGC R: ACGTTGGATGCCTGATGCCACCATAGAAAG	47.7
KCNN3	F: ACGTTGGATGCATGGCACCCAGAAAGTTAC R: ACGTTGGATGTGACTAACTCCTGTCTTCCC	51.1

### 2.4. Statistics Analysis

Microsoft Excel 2019 software was applied to the *BRCA1* genes g.42548851 C > T, g.42581575 T > C and g.42560830 A > T loci and g.77092130 G > A loci of *PPAP2B* gene and g.103522805 C > A loci of *KCNN3* gene in sheep. The genotype frequency, gene frequency, polymorphic information content (PIC), heterozygosity (He) and effective allele number (Ne) were counted and Hardy–Weinberg test was performed. The general linear model in SPSS 19.0 software was used to analyze the association between genotypes and lambing number phenotypes in Small‐Tailed Han sheep, and the results were expressed in the form of mean ± standard error.

## 3. Results

### 3.1. Population Genetic Analysis of SNPs in *BRCA1*, *PPAP2B*, and *KCNN3*

Three SNPs in *BRCA1* (g.42548851 C > T, g.42581575 T > C, and g.42560830 A > T) were detected (Table 2). Three genotypes were detected at all three loci, g.42548851 C > T including the base pairs CC, CT, and TT, g.42581575 T > C including TT, TC, and CC, and g.42560830 A > T including AA, AT, and TT. The g.42548851 C > T locus had moderate polymorphism (0.25 < PIC < 0.5) in Bamei mutton sheep but had low polymorphism (PIC < 0.25) in the other four sheep breeds; the Chi‐square test revealed that this locus was under Hardy–Weinberg equilibrium in all five sheep breeds (*p* > 0.05). The g.42581575 T > C locus had moderate polymorphism (0.25 < PIC < 0.5) in Bamei mutton sheep but had low polymorphism (PIC < 0.25) in the other four sheep breeds; in addition, this locus was under Hardy–Weinberg equilibrium (*p* > 0.05) in all sheep breeds except Hu sheep (*p* < 0.05). The g.42560830 A > T locus had moderate polymorphism (0.25 < PIC < 0.5) in Bamei mutton sheep but had low polymorphism (PIC < 0.25) in the other four sheep breeds; this locus was under Hardy–Weinberg equilibrium (*p* > 0.05) in all sheep breeds except Hu sheep (*p* < 0.05).

**Table 2:** Population genetic analysis of three loci of *BRCA1* in five sheep breeds.

Locus	Breed	Genotype frequency			Allele frequency		PIC	He	Ne	*Chi*‐square Test (*p* value)
g.42548851 C > T		**CC**	**CT**	**TT**	**C**	**T**				
	Small‐Tailed Han sheep	0.76	0.23	0.01	0.87	0.13	0.19	0.21	1.28	0.61
	Hu sheep	0.84	0.13	0.03	0.90	0.10	0.16	0.17	1.21	0.90
	Cele Black sheep	0.84	0.15	0.01	0.91	0.09	0.14	0.16	1.18	0.90
	Sunite sheep	0.84	0.16	0.00	0.92	0.08	0.07	0.12	1.14	0.71
	Bamei mutton sheep	0.23	0.44	0.33	0.45	0.55	0.37	0.50	1.98	0.53
g.42581575 T > C		**TT**	**TC**	**CC**	**T**	**C**				
	Small‐Tailed Han sheep	0.75	0.24	0.01	0.87	0.13	0.20	0.22	1.29	0.56
	Hu sheep	0.84	0.13	0.03	0.90	0.10	0.16	0.18	1.22	0.03
	Cele Black sheep	0.83	0.16	0.01	0.91	0.09	0.15	0.16	1.19	0.95
	Sunite sheep	0.84	0.16	0.00	0.92	0.08	0.14	0.15	1.17	0.71
	Bamei mutton sheep	0.14	0.46	0.40	0.37	0.63	0.36	0.47	1.87	0.99
g.42560830 A > T		**AA**	**AT**	**TT**	**A**	**T**				
	Small‐Tailed Han sheep	0.76	0.23	0.01	0.87	0.13	0.20	0.22	1.29	0.59
	Hu sheep	0.84	0.13	0.03	0.91	0.09	0.15	0.16	1.19	0.03
	Cele Black sheep	0.84	0.15	0.01	0.92	0.08	0.14	0.15	1.17	0.90
	Sunite sheep	0.84	0.16	0.00	0.92	0.08	0.14	0.15	1.17	0.71
	Bamei mutton sheep	0.23	0.44	0.33	0.45	0.55	0.37	0.49	1.98	0.53
										

One SNP in *PPAP2B* (g.77092130 G > A) and other one in *KCNN3* (g.103522805 C > A) were detected (Table 3). Three genotypes were detected at all two loci: g.77092130 G > A including the base pairs GG, GA, and AA and g.103522805 C > A including CC, CA, and AA. The g.77092130 G > A locus had moderate polymorphism (0.25 < PIC < 0.5) in Bamei mutton sheep but had low polymorphism (PIC < 0.25) in the other four sheep breeds; this locus was not under Hardy–Weinberg equilibrium in Cele Black sheep (*p* < 0.05) but was under in the other four breeds (*p* > 0.05). In addition, the g.103522805 C > A locus had moderate polymorphism (0.25 < PIC < 0.5) in Hu sheep but had low polymorphism (PIC < 0.25) in the other four sheep breeds; this locus was under Hardy–Weinberg equilibrium in Small‐Tail Han, Cele Black, Hu, and Sunite sheep (*p* > 0.05) but not in Bamei mutton sheep (*p* < 0.05).

**Table 3:** Population genetic analysis of two loci of PPAP2B and KCNN3 in five sheep breeds.

Locus	Breed	Genotype frequency			Allele frequency		PIC	He	Ne	*Chi*‐square test (*p* value)
g.77092130 G > A		**GG**	**AG**	**AA**	**G**	**A**				
	Small‐Tailed Han sheep	0.82	0.16	0.02	0.90	0.10	0.16	0.18	1.22	0.36
	Hu sheep	0.91	0.09	0.00	0.96	0.04	0.08	0.08	1.09	0.89
	Cele Black sheep	1.00	0.00	0.00	1.00	0.00	0.00	0.00	1.00	_
	Sunite sheep	0.85	0.14	0.01	0.92	0.08	0.14	0.15	1.72	0.54
	Bamei mutton sheep	0.16	0.84	0.00	0.58	0.42	0.37	0.49	1.95	0.71
g.103522805 C > A		**CC**	**CA**	**AA**	**C**	**A**				
	Small‐Tailed Han sheep	0.98	0.02	0.00	0.99	0.01	0.02	0.02	1.02	0.98
	Hu sheep	0.58	0.37	0.05	0.77	0.23	0.29	0.36	1.56	0.99
	Cele Black sheep	0.99	0.01	0.00	0.99	0.01	0.01	0.01	1.01	0.99
	Sunite sheep	0.96	0.04	0.00	0.98	0.02	0.04	0.04	1.04	0.98
	Bamei mutton sheep	0.00	0.00	1.00	1.00	0.00	0.00	0.00	1.00	_

The results showed that *BRCA1* and *PPAP2B* have three genotypes, but *KCNN3* only has two genotypes in Small‐Tailed Han sheep (Figure 1).

Figure 1Genotyping results of candidate SNPs of *BRCA1*, *PPAP2B*, and *KCNN3* genes using MassARRAY SNP system. *BRCA1* (A), *PPAP2B* (B), and *KCNN3* (C).

**Figure:**
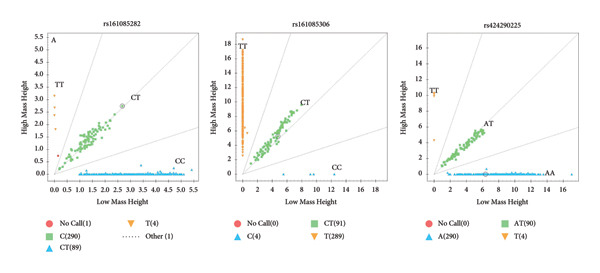
(A)

**Figure:**
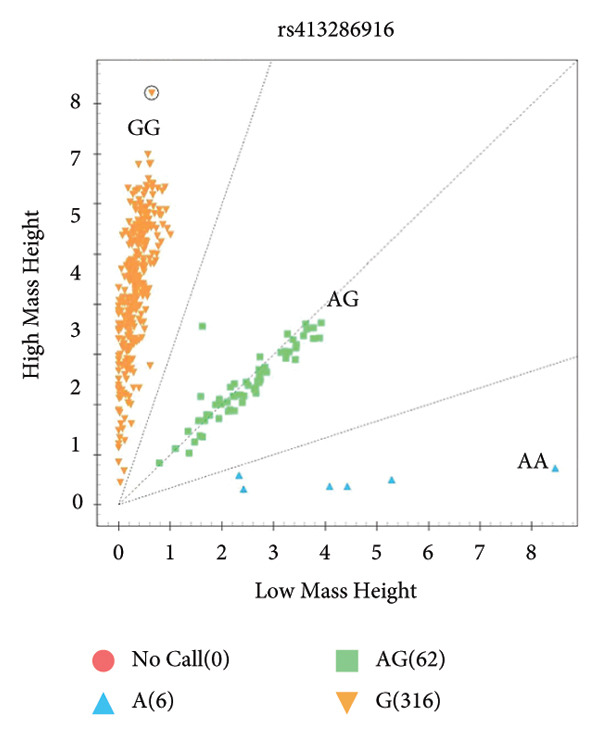
(B)

**Figure:**
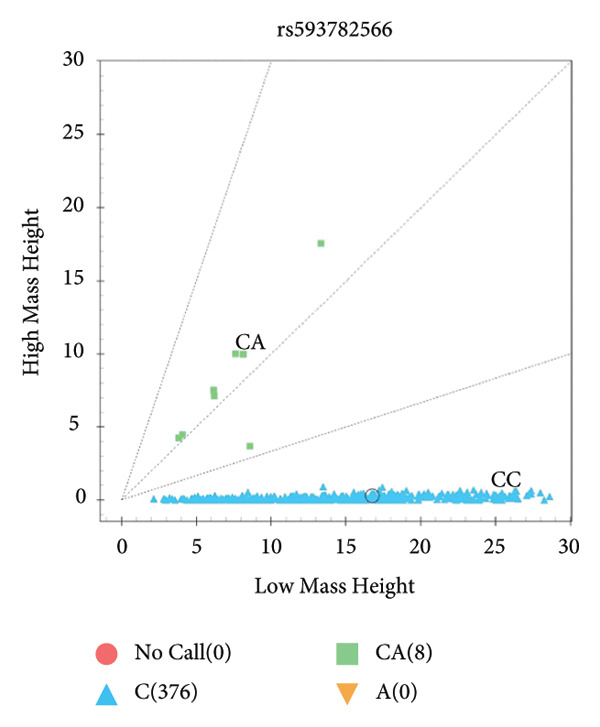
(C)

### 3.2. Association Analysis of Three SNPs With Litter Size in Small‐Tailed Han Sheep

To better understand the association of g.42548851 C > T, g.42581575 T > C, and g.42560830 A > T with litter size, an association analysis was performed for these three SNPs in terms of litter size in 384 Small‐Tailed Han sheep. There was no significant association between each genotype of the three loci and litter size in Small‐Tailed Han sheep. Except for the genotype where the litter size was not recorded, each genotype was successively increasing from the first to the third parity (Table 4).

**Table 4:** Analysis of different loci and litter size at *BRCA1* gene in Small‐Tailed Han sheep.

Locus	Genotype	Litter size		
		First parity (*N*)	Second parity (*N*)	Third parity (*N*)
g.42548851 C > T	CC	2.22 ± 0.05 (263)	2.45 ± 0.06 (203)	2.95 ± 0.12 (81)
	CT	2.18 ± 0.10 (79)	2.46 ± 0.11 (63)	2.88 ± 0.15 (16)
	TT	2.25 ± 0.484 (4)	2.33 ± 0.67 (3)	_
g.42581575 T > C	TT	2.22 ± 0.05 (262)	2.45 ± 0.06 (203)	2.95 ± 0.12 (81)
	TC	2.17 ± 0.09 (81)	2.46 ± 0.11 (63)	2.88 ± 0.15 (16)
	CC	2.25 ± 0.48 (4)	2.33 ± 0.67 (3)	_
g.42560830 A > G	AA	2.22 ± 0.05 (263)	2.45 ± 0.06 (203)	2.95 ± 0.12 (81)
	AT	2.18 ± 0.10 (80)	2.46 ± 0.11 (63)	2.88 ± 0.15 (16)
	TT	2.25 ± 0.48 (4)	2.33 ± 0.67 (3)	_

In the g. 77,092,130 G > A locus, there was no significant difference between the genotypes of the first and third fetuses (*p* > 0.05), but there was a significant difference between the different genotypes of the second fetuses of Small‐Tailed Han sheep, and there was a significant difference between the GG and AA genotypes, and the GG genotype was significantly higher than the AA genotype (*p* < 0.05) (Table 5).

**Table 5:** Analysis of different loci and litter size at *PPAP2B* gene in Small‐Tailed Han sheep.

Locus	Genotype	Litter size		
		First parity (*N*)	Second parity (*N*)	Third parity (*N*)
g.77092130 G > A	GG	2.19 ± 0.05 (286)	2.64 ± 0.06^a^ (214)	2.86 ± 0.11 (83)
	GA	2.38 ± 0.1 (56)	2.37 ± 0.12^ab^ (40)	3.33 ± 0.26 (12)
	AA	1.80 ± 0.20 (15)	1.75 ± 0.05^b^ (4)	4.00 ± 2.00 (2)

*Note:* Different small letters in the same group mean significant difference (*p* < 0.05).

At the g.103522805 C > A locus in the Small‐Tailed Han sheep, no significant differences in litter size at each parity were found among different genotypes, and only CC and CA genotypes had litter size record. However, the graph shows that the mean and standard error of the number of lambs produced in the latter litter of the same genotype is greater than that of the previous litter (Table 6).

**Table 6:** Analysis of different loci and litter size at *KCNN3* gene in Small‐Tailed Han sheep.

Locus	Genotype	Litter size		
		First parity (*N*)	Second parity (*N*)	Third parity (*N*)
g.103522805 C > A	CC	2.21 ± 0.05 (340)	2.46 ± 0.06 (221)	2.86 ± 0.11 (83)
	CA	2.38 ± 0.10 (56)	2.45 ± 0.13 (44)	3.33 ± 0.26 (12)

## 4. Discussion

### 4.1. The Relationship Between BRCA1 Gene Polymorphism and Sheep Reproductive Performance

The BMPR1B gene has been well established as a major genetic determinant influencing sheep reproductive traits [1]. Our findings confirm previous reports regarding the regulatory role of *BRCA1* in modulating BMPR1B expression in ovarian granulosa cells [20], highlighting the functional importance of this genetic interaction in sheep reproduction [21]. The observed moderate polymorphism (0.25 ≤ PIC < 0.5) at all three *BRCA1* loci (g.42581575 T > C, g.42560830 A > T, and g.42548851 C > T) specifically in Bamei mutton sheep suggests potential for selective breeding applications in this breed.

The Hardy–Weinberg equilibrium analysis revealed interesting breed‐specific patterns. While the g.42548851 C > T locus maintained equilibrium across all five studied breeds, the other two loci showed equilibrium in all breeds except Hu sheep. This deviation in Hu sheep may reflect unique selection pressures or population genetic characteristics in this breed. However, the lack of significant association between these *BRCA1* polymorphisms and litter size in Small‐Tailed Han sheep indicates that these specific variants may not be major determinants of prolificacy in this breed [22], suggesting the involvement of other genetic factors in its reproductive performance.

### 4.2. The Relationship Between KCNN3 Gene Polymorphism and Sheep Reproductive Performance

The *KCNN3* gene’s role in reproductive physiology, particularly during parturition, is supported by evidence from transgenic mouse models showing that proper regulation of *KCNN3* expression is essential for normal uterine contractions [15]. Our finding that the g.103522805 C > A locus displays moderate polymorphism exclusively in Hu sheep (0.25 ≤ PIC < 0.5) suggests this genetic variant may have particular relevance for this breed’s reproductive characteristics.

The Hardy–Weinberg equilibrium was maintained at this locus in all breeds except Bamei mutton sheep, mirroring the breed‐specific patterns observed with *BRCA1* polymorphisms. The absence of significant association between the *KCNN3* polymorphism and litter size in Small‐Tailed Han sheep parallels our *BRCA1* findings, reinforcing the concept that reproductive performance in this breed is likely influenced by a different set of genetic factors. The specific functional significance of the *KCNN3* polymorphism in Hu sheep warrants further investigation, particularly given this breed’s known reproductive characteristics [23].

### 4.3. The Relationship Between *PPAP2B* Gene Polymorphism and Sheep Reproductive Performance

The PPAP2B gene, encoding a lipid phosphatase involved in cellular signaling [24], has emerged as a potential regulator of sheep reproductive performance, particularly in relation to nonseasonal breeding traits [10]. Our study revealed that the *PPAP2B* gene exhibits distinct polymorphic patterns in Hu sheep, a breed known for its year‐round reproductive capability. The moderate polymorphism observed at specific loci (g.77092130 G > A) suggests that *PPAP2B* may contribute to the genetic basis of Hu sheep’s unique reproductive physiology.

The Hardy–Weinberg equilibrium analysis indicated that these *PPAP2B* variants were in equilibrium across most breeds except for Small‐Tailed Han sheep, which exhibited significant deviation (*p* < 0.05). This could imply selective pressures or genetic drift influencing *PPAP2B* allele frequencies in this breed. However, association analysis did not reveal a significant correlation between *PPAP2B* polymorphisms and litter size in Hu sheep, suggesting that while this gene may influence reproductive seasonality, it may not be a major determinant of prolificacy. The breed‐specific polymorphism patterns observed in Hu sheep highlight its potential as a candidate gene for improving nonseasonal reproduction in sheep breeding programs. Further functional validation, including gene expression and knockout studies, will be essential to clarify its precise role in sheep reproductive biology.

## 5. Conclusions

This study presents the inaugural analysis of polymorphism at a locus within *PPAP2B* and posits that this locus (g.77092130 G > A) exhibits a significant correlation with the number of lambs produced in the second litter of Small‐Tailed Han sheep. These data provide valuable genetic markers for sheep breeding.

## Ethics Statement

All the experimental procedures mentioned in the present study were approved by the Science Research Department (in charge of animal welfare issues) of Institute of Animal Science (IAS‐ CAAS) (Beijing, China). Also, ethical approval was given by the Animal Ethics Committee of the IAS (IAS2021‐24).

## Conflicts of Interest

The authors declare no conflicts of interest.

## Author Contributions

Yu He reformed the experiments, analyzed data, and wrote the first draft. Xiangyu Wang contributed to the tissue, serum collection, and pretreatment of the samples. Chen Liang provided modification suggestions. Mingxing Chu contributed to the experimental design and manuscript revision.

## Funding

This study was funded by the Central Public‐Interest Scientific Institution Basal Research Fund (Y2024YJ08), the China Agriculture Research System of MOF and MARA (CARS‐38), and the Agricultural Science and Technology Innovation Program of China (ASTIP‐IAS13).

## Data Availability

The data that support the findings of this study are available from the corresponding author upon reasonable request.
